# The study of Wilson disease in pregnancy management

**DOI:** 10.1186/s12884-019-2641-8

**Published:** 2019-12-26

**Authors:** Xu-En Yu, Min Pan, Yong-Zhu Han, Ren-Min Yang, Juan Wang, Shan Gao

**Affiliations:** 1 Department of Pharmacology, Basic Medical College, Anhui Medical University, Hefei, 230032 China; 2 Affiliated Hospital of the Institute of Neurology, Anhui University of Chinese Medicine, Hefei, 230061 China; 3 Department of Neurology, Hefei Second People’s Hospital, Hefei, 230001 China

**Keywords:** Copper, Pregnancy, Spontaneous abortion, Wilson disease

## Abstract

**Introduction:**

Pregnancy management in women with Wilson disease (WD) remains an important clinical problem. This research was conducted to investigate how to avoid worsening of WD symptoms during pregnancy and increase pregnancy success in women with WD by identifying the best pregnancy management approaches in these patients.

**Patients and methods:**

The clinical data of 117 pregnancies among 75 women with WD were retrospectively analyzed. Related information of the fetus was also recorded and analyzed. At the same time, regression analysis was performed for data of 22 pregnant women without WD, as normal controls.

**Results:**

Of a total of 117 pregnancies among the 75 women with WD and 31 pregnancies among the 22 control womenincluded in this study, there were 108 successful pregnancies and 9 spontaneous abortions. Among the 108 successful pregnancies, 97 women a history of copper chelation therapy before pregnancy; all 97 women stopped anti-copper therapy during pregnancy. The nine women with spontaneous abortion had no pre-pregnancy history of copper displacement therapy. The incidence of lower limb edema was higher in the WD group than in normal controls (*P* = 0.036). Compared with the control group, there was a higher proportion in the WD group of male infants (*P* = 0.022) and lower average infant birth weight (t = 3.514, *P* = 0.001).

**Conclusion:**

It is relatively safe for women with WD patients to become pregnant. The best management method for pregnancy in women with WD may be intensive pre-pregnancy copper chelation therapy and no anti-copper treatment during pregnancy.

## Introduction

Wilson disease (WD) is an autosomal recessive genetic disorder that can be treated at present [1]. WD is caused by excessive deposition of ATP7B copper transporter protein, which causes a variety of symptoms in different organs [2]. The most common copper deposition sites are the liver and the brain [1]. Currently, the main treatment method involves using copper chelating agents, such as penicillamine and trientine [3–5]. Few studies have reported on pregnancy in patients with WD, and the findings of the existing research vary considerably. It has been reported that symptoms of liver and brain damage are significantly aggravated during pregnancy in women with WD [6–9], but a large number of successful pregnancies have also been reported in these patients [10–15]. Research on pregnancy in WD is of great importance not only to clinicians who treat these patients but also to women with WD who are pregnant or planning to become pregnant [16]. Therefore, a retrospective analysis was conducted of pregnant women with WD in China, focusing on how to avoid the aggravation of disease symptoms during pregnancy and increase the pregnancy success rate of women with WD, to find the best management method for this patient population.

## Material and methods

### Patients

We collected the clinical data of 75 female patients with WD and with previous pregnancy experience (a total of 117 pregnancies), who were admitted to the affiliated hospital of the Institute of Neurology, Anhui University of Chinese Medicine from January 2014 to December 2018. All patients with WD enrolled in this study met the diagnostic criteria of WD [16, 17]. In addition, 22 pregnant women without WD were selected as normal controls, with 31 pregnancies that originated in Hefei Maternal and Child Health Care Hospital; the controls were age-matched with those in the case group (t = 0.352, *P* = 0.728). Additionally, the subjects who had abnormal examinations before pregnancy were excluded. This study was approved by the ethics committee of Anhui University of Chinese Medicine and all participants signed informed consent.

### Research methods

The clinical data of the 117 pregnancies among the 75 women with WD were retrospectively analyzed before, during, and after pregnancy; relevant data of the fetus were also statistically analyzed. In addition, the pregnancy course and pregnancy outcome of the 31 pregnancies in the 22 control patients were also reviewed.

### Statistical methods

Quantitative data consistent with a normal distribution are described as mean ± standard deviation (SD), and the pregnancy status of women in the WD and control groups was compared using a t-test of two independent samples. Sample size and percentage (%) were used to describe the classified data, and the chi-square test (or Fisher exact probability method) was used to compare the two groups. All statistical analyses were performed using IBM SPSS 23.0 (IBM Corp., Armonk, NY, USA), and *P* < 0.05 (two-tailed) was considered statistically significant.

## Results

### Clinical data of patients with WD

Among the 75 enrolled women with WD, a total of 117 pregnancies were recorded. The age at childbirth of women with WD was 22–34 years, with an average age of 27.72 ± 2.79 years, and the age at hospitalization for symptoms of WD was 24–36 years old, with an average age 30.43 ± 2.99 years. In the 75 women with WD, the total 117 pregnancies resulted in 108 successful pregnancies and 9 spontaneous abortions. Among these, 97 of the 108 women with successful pregnancies had a history of hospitalization and intensive copper displacement therapy before pregnancy; these 97 women stopped taking anti-copperdrugs during pregnancy. The nine women who had spontaneous abortion had no history of copper displacement therapy before pregnancy. Changes were observed in the pregnancy condition of the included women with WD. Among 21 pregnancies, 17 with WD had obvious liver injuries. In addition, 10 women with WD (in 10 pregnancies) had severe neurological symptoms.

### Comparison of pregnancy complications between normal patients and those with WD

There were 117 and 31 pregnancies among the 75 patients with WD and 22 control patients, respectively, which were matched according to age in the analysis. Postpartum complications and pregnancy complications in the two groups were compared. Lower extremity edema was the main postpartum complication, and the difference was statistically significant (χ^2^ = 10.482, *P* = 0.036), as shown in Table 1.

**Table 1:** Pregnancy complications in normal controls and women with Wilson disease

Group (n)	Age at pregnancy (y), mean ± SD	Gravida	Pregnancy outcomes, n (%)		Complications, n (%)						Pregnancy complications (%)		
			Success	Failure	Extremity edema	Jaundice	Ascites	Hyper-tension	Hyper-glycemia	Hepatic injury	Premature rupture of membranes	Hydramnios	Oligohydramnios
WD group (75)	27.72 ± 2.79	117	108 (92.3)	9 (7.7)	105 (78.4)	3 (2.2)	3 (2.2)	3 (2.2)	2 (1.5)	18 (13.4)	5 (50.0)	2 (20.0)	3 (30.0)
Control group (22)	27.41 ± 3.85	31	30 (96.8)	1 (3.2)	30 (81.1)	0 (0)	0 (0)	0 (0)	5 (13.5)	2 (5.4)	2 (66.7)	1 (33.3)	0 (0)
t/χ^2^	0.352^a^		0.229^b^		10.482^c^						1.229^c^		
P	0.728		0.632		0.036						1.000		

^a^ t-value

^b^ Continuously corrected chi-square value

^c^ Chi-square value of Fisher’s exact test; mean ± SD

### Childbirth in normal controls and women with WD

The proportion of male infants was higher among women with WD than that among women in the control group (χ^2^ = 5.249, *P* = 0.022). The average infant birth weight in the WD group was lower than that in the control group, and the difference was statistically significant (t = 3.514, *P* = 0.001). However, there was no statistically significant difference between the two groups in terms of natural delivery and fetal Apgar score, as shown in Table 2.

**Table 2:** Data of childbirth in women with Wilson disease compared with normal women

Group (n)	Sex (%)		Delivery mode (%)		Fetal birth data (mean ± SD)	
	Male	Female	Natural delivery	Cesarean delivery	Apgar score	Weight (kg)
WD group (108)	65 (60.2)	43 (39.8)	67 (62.0)	41 (38.0)	9.88 ± 0.33	3.41 ± 0.40
Controlgroup(30)	11 (36.7)	19 (63.3)	13 (43.3)	17 (56.7)	9.87 ± 0.35	3.64 ± 0.29
χ^2^/t	5.249^a^		3.371^a^		0.145^b^	3.514^b^
P	0.022		0.066		0.885	0.001

^a^Chi-square value

^b^ t-value; mean ± SD

## Discussion

WD is an autosomal recessive hereditary disease that can be treated at present [3, 4]. Many patients with WD disease are women of childbearing age who are diagnosed with early-stage disease [17, 18]. Some women with WD become pregnant after disease onset and diagnosis; therefore, further research on the management of pregnant patients with WD is urgently needed. This retrospective analysis was conducted, using data of pregnant women with WD in China, focusing on several aspects and comparing choices in pregnancy, to better guide women of childbearing age who have WD.

In the present retrospective analysis of 117 pregnancies in 75 women with WD, there were 108 successful pregnancies (92.3%) and successful deliveries. The vast majority of the patients included in this study had a history of pre-pregnancy hospitalization in which they received treatment with copper chelation therapy and disease evaluation during the first half of their pregnancy. These results are not completely consistent with the literature [15, 19]. Therefore, it is strongly recommended that women with WD need systematic evaluation and treatment during pre-pregnancy. Studies have found that the clinical effects of WD on pregnancy outcomes mainly include neurological symptoms in pregnant women, as well as spontaneous abortion [7, 8, 19]. The data analyzed in the present study showed that neurological symptoms were more frequent among women with WD than spontaneous abortions or liver and bone damage. These findings are consistent with the results of the present analysis, in which all women with spontaneous abortion had no prior history of anti-copper therapy.

Analysis of the present data showed that hospitalized women with WD received copper displacement treatment and disease evaluation before their pregnancy; these patients stopped taking copper displacement medications during pregnancy. This finding is inconsistent with reports in the recent literature [13, 14]; additional multicenter studies, are needed to clarify this issue. The authors believe that clinical symptoms in these patients are relatively reduced in pregnancy, which could be related to the normal metabolism of copper by the fetus. This result also found that patients with hospital readmission after childbirth for symptoms of liver injury gradually recovered after copper chelation treatment. However, recovery was slower after anti-copper treatment in patients readmitted to the hospital after childbirth with aggravated neurological symptoms. These findings are in line with those of recent reports [7, 19]. The aggravation of neurological symptoms in women with WD during pregnancy has been widely reported [6, 7, 19], but the specific mechanism has not been further explored. The present data analysis indicated that neurological symptoms were aggravated in 10 pregnant women with WD, and 24-h urine monitoring showed that copper levels were not very high. Therefore, aggravation of neurological symptoms in patients with WD may not be completely consistent with excessive copper deposition in the body, which differs considerably from published reports [6, 7, 19].

Many researchers have stated that the most important factor influencing the pregnancy outcome of women with WD is continuous copper displacement therapy, and that continuous treatment is the best approach to avoid the aggravation of the disease and increase the success rate of pregnancy [10–15, 20]. The authors believe that the pre-pregnancy copper displacement treatment and condition assessment, followed by suspension of drug therapy during pregnancy is the best way to avoid aggravated disease symptoms during pregnancy and to increase the success rate of pregnancy in women with WD.

In our study population, women with WD were more likely to have complications of lower limb edema during pregnancy than normal pregnant women, and more women in the WD group had male infants and infants with lower birth weight than women in the control group, there was no difference in the mode of delivery or fetal Apgar score between the patient groups. However,the subjects who had abnormal examinations during pregnancy were excluded may contribute to the limited sample size, especiallythe control group, so whether our findings can comprehensively reflect the pregnancy status of women with WD requires confirmation in future studies with larger sample size.

## Conclusions

To sum up, women with WD have complicated issues in pregnancy, and prospective studies in this patient population are lacking; all published reports are retrospective analyses. It is relatively safe for women with WD patients to become pregnant. The best management method for pregnancy in women with WD may be intensive pre-pregnancy copper chelation therapy and no anti-copper treatment during pregnancy.

## Data Availability

The datasets are available from the corresponding author on reasonable request.

## Abbreviations

SD: Standard Deviation
WD: Wilson Disease
